# Supplementary material for: Multi‐dataset identification of innovative feature genes and molecular mechanisms in keratoconus
Source: J Cell Mol Med. 2024 Sep 19;28(18):e70079. doi: 10.1111/jcmm.70079 (PMC11412914; doi:10.1111/jcmm.70079)
Supplement: Supplementary file 2 — Material S2. [file JCMM-28-e70079-s002.zip › 7.GSEA analysis/3.DBNDD1/KEGG_DNA_REPLICATION.html]

Details for gene set KEGG\_DNA\_REPLICATION[GSEA]

|  || Dataset | DBNDD1.group.cls#h\_versus\_l |
| Phenotype | group.cls#h\_versus\_l |
| Upregulated in class | l |
| GeneSet | KEGG\_DNA\_REPLICATION |
| Enrichment Score (ES) | -0.4635459 |
| Normalized Enrichment Score (NES) | -1.5141948 |
| Nominal p-value | 0.024242423 |
| FDR q-value | 0.78473055 |
| FWER p-Value | 0.776 |
Table: GSEA Results Summary

  

Fig 1: Enrichment plot: KEGG\_DNA\_REPLICATION      
 Profile of the Running ES Score & Positions of GeneSet Members on the Rank Ordered List

  

| SYMBOL | TITLE | RANK IN GENE LIST | RANK METRIC SCORE | RUNNING ES | CORE ENRICHMENT || 1 | RPA4 | na | 2636 | 0.266 | 0.0188 | No |
| 2 | RPA2 | na | 11249 | 0.055 | -0.2098 | No |
| 3 | SSBP1 | na | 11481 | 0.052 | -0.1978 | No |
| 4 | RNASEH2B | na | 12867 | 0.039 | -0.2237 | No |
| 5 | POLD2 | na | 14464 | 0.027 | -0.2602 | No |
| 6 | RPA1 | na | 14916 | 0.023 | -0.2649 | No |
| 7 | POLD4 | na | 15345 | 0.020 | -0.2700 | No |
| 8 | POLE4 | na | 15472 | 0.019 | -0.2667 | No |
| 9 | POLE | na | 16056 | 0.015 | -0.2781 | No |
| 10 | PCNA | na | 16348 | 0.014 | -0.2816 | No |
| 11 | POLE3 | na | 18619 | -0.002 | -0.3465 | No |
| 12 | POLD1 | na | 19928 | -0.012 | -0.3798 | No |
| 13 | DNA2 | na | 20332 | -0.016 | -0.3857 | No |
| 14 | RNASEH1 | na | 20676 | -0.019 | -0.3889 | No |
| 15 | POLD3 | na | 21651 | -0.028 | -0.4068 | No |
| 16 | POLA2 | na | 22961 | -0.043 | -0.4294 | No |
| 17 | RNASEH2A | na | 24145 | -0.058 | -0.4428 | Yes |
| 18 | POLA1 | na | 24523 | -0.064 | -0.4310 | Yes |
| 19 | RPA3 | na | 24548 | -0.064 | -0.4089 | Yes |
| 20 | RFC2 | na | 24735 | -0.067 | -0.3905 | Yes |
| 21 | MCM3 | na | 24829 | -0.068 | -0.3689 | Yes |
| 22 | LIG1 | na | 25137 | -0.073 | -0.3518 | Yes |
| 23 | RFC5 | na | 25224 | -0.074 | -0.3280 | Yes |
| 24 | RFC4 | na | 25689 | -0.082 | -0.3123 | Yes |
| 25 | FEN1 | na | 25819 | -0.084 | -0.2861 | Yes |
| 26 | PRIM2 | na | 26139 | -0.090 | -0.2631 | Yes |
| 27 | MCM7 | na | 26354 | -0.095 | -0.2356 | Yes |
| 28 | RFC1 | na | 26544 | -0.098 | -0.2060 | Yes |
| 29 | RFC3 | na | 26553 | -0.099 | -0.1711 | Yes |
| 30 | RNASEH2C | na | 27207 | -0.113 | -0.1499 | Yes |
| 31 | MCM4 | na | 27439 | -0.117 | -0.1148 | Yes |
| 32 | MCM2 | na | 28139 | -0.134 | -0.0872 | Yes |
| 33 | MCM5 | na | 28262 | -0.137 | -0.0420 | Yes |
| 34 | PRIM1 | na | 29087 | -0.160 | -0.0089 | Yes |
| 35 | MCM6 | na | 29866 | -0.184 | 0.0342 | Yes |
| 36 | POLE2 | na | 32556 | -0.298 | 0.0626 | Yes |
Table: GSEA details [plain text format]

  

Fig 2: KEGG\_DNA\_REPLICATION      
 Blue-Pink O' Gram in the Space of the Analyzed GeneSet

  

Fig 3: KEGG\_DNA\_REPLICATION: Random ES distribution      
 Gene set null distribution of ES for **KEGG\_DNA\_REPLICATION**
